# In Silico Structural Protein Evaluation of the Phenylalanine Hydroxylase p.(Tyr77His) Variant Associated with Benign Hyperphenylalaninemia as Identified through Mexican Newborn Screening

**DOI:** 10.3390/children10121865

**Published:** 2023-11-28

**Authors:** Marcela Vela-Amieva, Miguel Angel Alcántara-Ortigoza, Ariadna González-del Angel, Isabel Ibarra-González, Liliana Fernández-Hernández, Sara Guillén-López, Lizbeth López-Mejía, Cynthia Fernández-Lainez

**Affiliations:** 1 Laboratorio de Errores Innatos del Metabolismo y Tamiz, Instituto Nacional de Pediatría, Secretaría de Salud, Ciudad de México 04530, Mexico; mvelaa@pediatria.gob.mx (M.V.-A.); guillen.lopez.sara@gmail.com (S.G.-L.); lizbeth712@hotmail.com (L.L.-M.); 2 Laboratorio de Biología Molecular, Instituto Nacional de Pediatría, Secretaría de Salud, Ciudad de México 04530, Mexico; malcantaraortigoza@gmail.com (M.A.A.-O.); ariadnagonzalezdelangel@gmail.com (A.G.-d.A.); dralilianafernandez@gmail.com (L.F.-H.); 3 Centro de Alta Especialidad en Genética Humana DNA-GEN S.C., Ciudad de México 14070, Mexico; 4 Unidad de Genética de la Nutrición, Instituto de Investigaciones Biomédicas UNAM, Ciudad de México 04510, Mexico; icig@servidor.unam.mx

**Keywords:** inborn errors of intermediary metabolism, amino acids, aminoacidopathies, intellectual disability, rare diseases, Cuban PKU population, Mexican PKU population

## Abstract

Hyperphenylalaninemia (HPA), which includes phenylketonuria (PKU), is a genetic autosomal recessive disorder arising from a deficiency in the enzyme named phenylalanine hydroxylase (PAH). Affected patients can experience severe and irreversible neurological impairments when phenylalanine (Phe) blood concentration exceeds 360 μmol/L (6 mg/dL). Here, we describe a female HPA patient who was born in Mexico to Cuban non-consanguineous parents and identified by newborn screening, and who bears the previously unreported *PAH* NM_000277.3(PAH):c.[229T>C];[1222C>T] or p.[Tyr77His];[Arg408Trp] genotype. At diagnosis, the patient showed a Phe blood level of 321 μmol/L (5.3 mg/dL), indicative of mild HPA. Neither of the *PAH* variants found in this patient had been previously reported in the mutational *PAH* spectrum of the Mexican population. The c.229T>C or p.(Tyr77His) *PAH* variant was previously related to mild HPA in the Swedish population. Our in silico structural analysis and molecular docking showed that mutated His 77 residue is located in the allosteric site of PAH at the interface of the two monomers. The PDBsum in silico tool predicted that this variant would cause minimal structural disturbance of the protein interface in the presence of Phe at the allosteric site. Docking studies revealed that these structural changes might be attenuated by the allosteric effect of Phe. Given the classic PKU phenotype conditioned by the “Celtic” or c.[1222C>T] or p.(Arg408Trp) *PAH* variant, which is the second variant in this patient, we propose that p.(Tyr77His) has a hypomorphic feature that could explain her mild HPA phenotype. Our results show the importance of following up on cases detected by NBS and the value of genetic studies and in silico tools that aid in the establishment of correct therapeutic strategies.

## 1. Introduction

**Figure 1:**
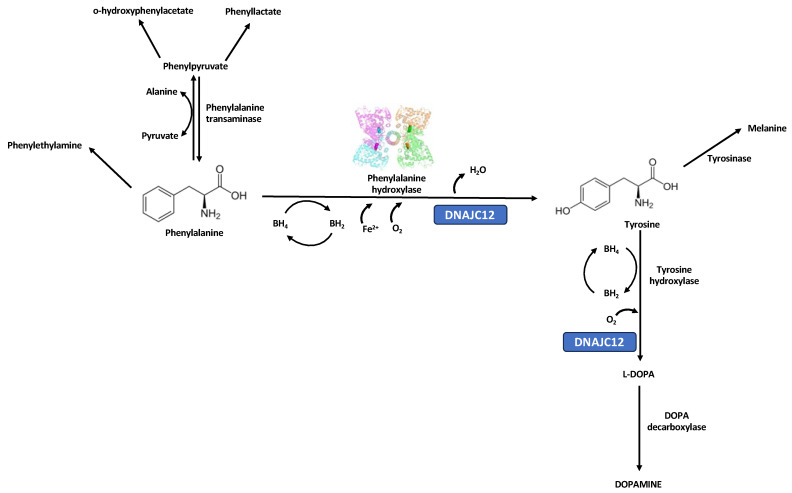
The PAH enzyme catalyzes the hydroxylation of phenylalanine (Phe) to tyrosine (Tyr) in the presence of iron, molecular oxygen, the cofactor tetrahydrobiopterin (BH4), and the molecular chaperone HSP70 family member, DNAJ/HSP40 homolog, subfamily C, member 12 (also called DNAJC12; OMIM*606060). One atom of molecular oxygen binds to the benzene ring of Phe to give Tyr, while the other atom is reduced to water. On each catalysis, BH4 is oxidized to dihydrobiopterin (BH2). PAH deficiency leads to the toxic accumulation of Phe and its derivatives (phenylpyruvate, hydroxy-phenylacetate, phenyllactate) in various tissues, particularly in the brain; this causes irreversible cognitive and neurological impairments if left untreated [5]. Deficiencies related to synthesizing and/or recycling the BH4 or DNAJC12 chaperone are inherited mainly as autosomal recessive traits and are associated with less frequent types of *PAH*-nonrelated hyperphenylalaninemia. The different colors in the figure of PAH enzyme denote the four different subunits that constitute the homotetramer.

Hyperphenylalaninemia (HPA) is an autosomal recessive disorder arising from deficiency of phenylalanine hydroxylase (PAH; EC1.14.16.1), which is an enzyme crucial for the conversion of the amino acid phenylalanine (Phe) to tyrosine (Tyr) (Figure 1). The spectrum of HPA includes phenylketonuria (PKU; OMIM#261600). Among inherited metabolic disorders, phenylketonuria (PKU) is a paradigmatic example for its historical, scientific, and clinical significance, especially as a biochemical cause of intellectual disability that can be prevented with early diagnosis and dietary treatment [1]. 

As occurs for other monogenic diseases, PAH deficiency shows a broad clinical spectrum that mainly depends on the degree to which the pathogenic variants in the *PAH* gene (12q23.2, OMIM*612349) cause structural and functional damage to the encoded enzyme [2,3]. PAH can range from clinically benign and mild forms to severe classical PKU, where practically no enzyme activity is detected. In the absence of treatment, PKU is characterized by microcephaly; hypopigmentation of hair, skin, and iris; neurological manifestations (limb spasticity, tremor, seizure disorder, severe intellectual disability); and behavioral disturbances, such as aggression, hyperactivity, anxiety, and social withdrawal [4].

In Mexico, newborn screening (NBS) for metabolic diseases was first introduced in 1973 by Dr. Antonio Velázquez [6,7]; this milestone initiative represented a pioneering program of its kind in Latin America. 

Despite the health system is highly fragmented in Mexico; currently, the official NBS panel of the Mexican Ministry of Health includes congenital hypothyroidism, congenital adrenal hyperplasia, galactosemia, cystic fibrosis, glucose-6-phosphate deficiency, and PKU [7]. A recent report indicated that PKU has a birth prevalence of one in 27,546 screened Mexican newborns [7]. 

The primary biochemical marker of PAH deficiency is Phe blood concentration, which is used to classify the disease along a range of mild HPA (MHP, pre-treatment blood Phe 120–600 μmol/L or 2–10 mg/dL), mild PKU (mPKU, pre-treatment blood Phe 600–1200 μmol/L or 10–20 mg/dL), and classical PKU (pre-treatment blood Phe > 1200 μmol/L or >20 mg/dL). The Phe level is considered along with the Tyrosine (Tyr) level and the Phe/Tyr ratio [8]. 

At present, molecular screening studies for some conditions, such as HPA, are recommended as second-line studies to confirm positive cases. However, the NBS field is evolving quickly, and some groups advocate that genomic sequencing of newborns be performed as a primary test [9]. 

It is generally accepted that MHP forms do not require treatment since Phe blood levels remain between 120 and 360 μmol/L (2 and 6 mg/dL) throughout life. However, it is not necessarily simple to group such patients in clinical practice. Sometimes other classification criteria must be used, such as dietary Phe tolerance, as Phe levels can vary along a lifespan depending on dietary intake and stressful factors [10,11]. 

Genotyping is necessary to determine phenotype/genotype correlations, understand the residual enzyme activity of each allele and genotype, and predict the response to sapropterin [2]. To date, 2209 *PAH* variants have been registered in the BioPKU database (BIOPKUdb) [12,13]. Extensive studies of the worldwide genotypic *PAH* spectrum have revealed some distinctive mutational distributions according to ancestry, especially in North American and European populations [8]. In the Mexican population, the mutational spectrum of *PAH* comprises 60 variants recently described from the largest Mexican cohort reported to date (124 unrelated HPA patients) [14]. Among the four most frequent alleles described, the p.(Val388Met) [rs62516101], c.1066-11G>A [rs5030855], and c.441+5G>T [rs62507321] variants showed allelic frequencies similar to those observed for other Latin American countries and even Spain, which could correlate with historical colonization [15]. Notably, the Mexican population also exhibited a unique predominance of the rare PKU-causing allele c.60+5G>T [rs62514895] (14.5% vs. 0.32% worldwide) but lacked the p.(Arg408Trp) or “Celtic” variant, which is considered the most frequent worldwide PKU-causing allele, especially in central and eastern Europe (44.4–53.7%) [8,14]. The remarkably uncommon p.(Tyr77His) variant described for the first time as a mild HPA-causing *PAH* allele in the Swedish population [12,16] was not previously identified in this large Mexican cohort [14].

Herein, we describe the clinical and biochemical phenotype of an HPA-affected female newborn of Cuban ancestry detected through the NBS program of the Mexican Ministry of Health who bears the previously unreported *PAH* genotype NM_000277.3(PAH): c.[229T>C];[1222C>T] or p.[Tyr77His];[Arg408Trp].

Since the global frequency of the p.(Tyr77His) variant is currently unknown [12], this is its first identification in Mexico, and the literature lacks any prediction of its structural effects on the encoded enzyme, we performed in silico structural analysis of the p.(Tyr77His) *PAH* variant in an effort to predict its effect and explore a possible genotype/phenotype correlation.

## 2. Materials and Methods

### 2.1. Patient Description

The patient was a newborn female, the first child of a 28-year-old mother and 31-year-old father who were healthy, non-consanguineous, and born in Holguin, Cuba. The mother reported adequate prenatal medical control during pregnancy, with folic acid and vitamin consumption initiated during the first weeks. She denied teratogen exposure but reported having had two urinary infections managed with antibiotic therapy during pregnancy. The patient was born through elective Cesarean section at 38 weeks of gestational age with a weight of 3100 g, length of 48 cm, and Apgar scores of 9 and 9 at one and five minutes, respectively. The patient was breastfed and discharged as a healthy newborn. The above-mentioned NBS was performed at 48 h of extrauterine life and showed a Phe blood concentration of 213 μmol/L (3.5 mg/dL). A second screening showed a Phe blood concentration of 435 μmol/L (7 mg/dL). Thus, at 28 days old, she was referred to our institution for confirmatory diagnosis and medical follow-up. Her Phe blood concentration at confirmatory diagnosis was 321 μmol/L (5.3 mg/dL). After two follow-up determinations of Phe blood concentration, the patient presented a slight increase above the desired level. Thus, a small amount of Phe-free formula was prescribed to complement the breastfeeding. At this time, the patient’s physical examination was within normal limits for her age (1 month and 11 days). No cutaneous rash, pale skin, or light hair was observed. Neurological exploration showed normal reflexes and muscular tone, and no abnormal movement was observed. Neurodevelopmental skills were appropriate for age.

### 2.2. Biochemical Confirmatory Analysis

Phe and Tyr blood concentrations were measured by high-performance liquid chromatography (HPLC) according to the methodology described by Hill [17].

### 2.3. PAH Genotype Analysis

For diagnostic confirmation of HPA, the patient’s genomic DNA was obtained from peripheral leukocytes using a saline precipitation method (Gentra Puregene Blood Kit, Gentra Systems, Minneapolis, MN, USA). The 13 coding *PAH* exons and their exon–intron borders were amplified with polymerase chain reaction (PCR) and subjected to automated bidirectional Sanger sequencing as previously described [14]. The variants found were classified according to BioPKUdb [12] and the literature. To confirm the patient´s compound heterozygous *PAH* genotype, genomic DNA samples were obtained from both parents and processed as described above.

### 2.4. In Silico Protein Modeling, Docking, and Mutational Analyses of p.(Tyr77His) PAH Variant 

The p.(Arg408Trp) variant, which involves alteration of a residue located at the catalytic site of the PAH enzyme, is a well-known variant that is associated with severe forms of the disease and leads to production of a protein with only 2% residual enzymatic activity. Since this variant formed part of the genotype of the current patient, who showed a benign HPA phenotype, it was assumed that the p.(Tyr77His) variant may encode a relatively functional protein; thus, we focused on modeling only the p.(Tyr77His) variant.

In silico protein modeling was used to obtain the tertiary structure of native PAH, localize the involved amino acid residues, and analyze the protein changes arising from the amino acid substitutions. All this was performed using the available crystallographic structures of human PAH (Protein Data Bank (PDB) codes: 6HYC [18] and 5FII [19]) and PyMOL software version 2.3.5 [20]. The PDBsum web server of the European Bioinformatics Institute [21] was used to analyze the interface interactions of the PAH tetramer in its wild-type (Wt) and p.(Tyr77His) mutant forms. Before the analysis, the energy of the protein was minimized using the Yasara minimization server [22].

In addition to binding at the catalytic site of PAH, Phe can bind an allosteric site on this protein [19]. Thus, to predict if the variants altered the binding of Phe to its allosteric site, after performing mutagenesis in silico, we conducted molecular docking in the crystallographic structure of this PAH domain (PDB code:5FII). To this end, we used the web service from the Molecular Modeling Group of the Swiss Institute of Bioinformatics (Lausanne, Switzerland) [23]. Phe was defined as the ligand, and the Wt and p.(Tyr77His) forms of PAH were defined as protein targets. 

## 3. Results 

**Figure 2:**
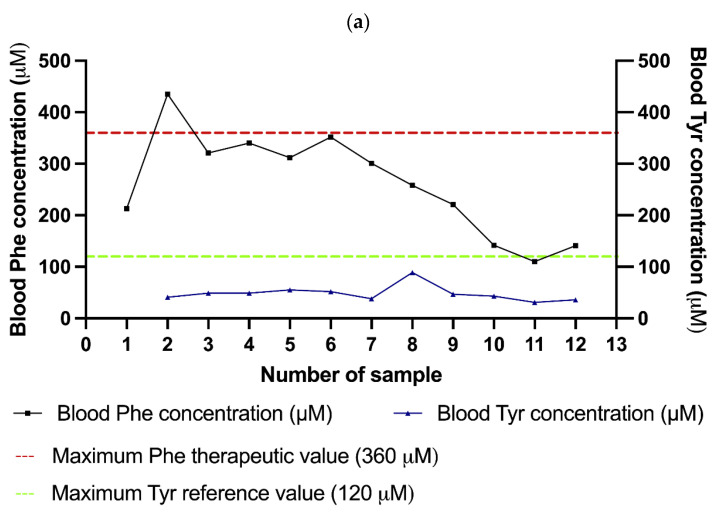

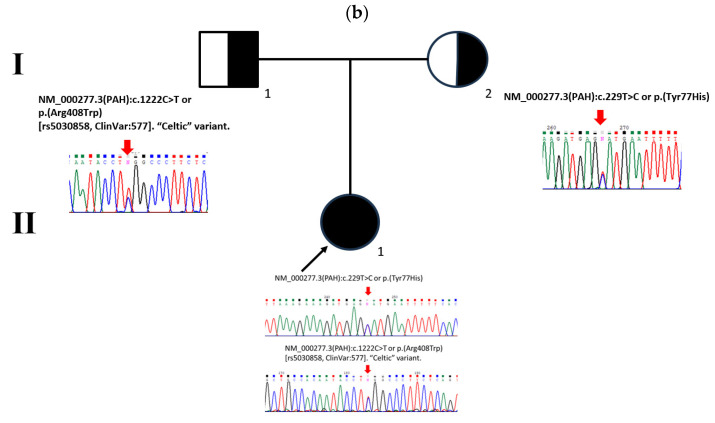
(**a**) Patient´s Phe and Tyr blood concentrations at diagnosis and follow-up. (**b**) Patient´s genealogy and the identified *PAH* genotypes. The paternal and maternal origin for the Celtic and p.(Tyr77His) variants, respectively, were confirmed in II-1. The carrier status in both parents confirms the compound heterozygous NM_000277.3(PAH): c.[229T>C];[1222C>T] or p.[Tyr77His];[Arg408Trp] genotype in II-1.

When the patient was 1 month and 11 days old, her follow-up HPLC test showed that the patient had a Phe blood concentration of 352 μmol/L (5.8 mg/dL), a Tyr blood concentration of 52 μmol/L (0.94 mg/dL), and a Phe/Tyr ratio of 6.7. Since her Phe level was close to 360 μmol/L (6 mg/dL), a mild supplementation of Phe-free metabolic formula was prescribed (0.4 g/kg/d of protein from 15 g of Anamix Infant™ formula plus ad libitum breastfeeding) to prevent an increase of the Phe level induced by potential catabolic events such as fever or infection. Her blood Phe levels gradually decreased thereafter (Figure 2a), so the formula prescription was reduced to 12 g per day (0.2 g/kg/d of protein), where it remains at present (7 months old). Biochemical evaluations of the patient and identification of a compound heterozygous NM_000277.3(PAH): c.[229T>C];[1222C>T] or p.[Tyr77His];[Arg408Trp] genotype (Figure 2b) collectively confirmed the *PAH*-related MHP diagnosis. The main characteristics of both variants, including the allelic phenotype values (APVs), are presented in Table 1.

**Figure 3:**
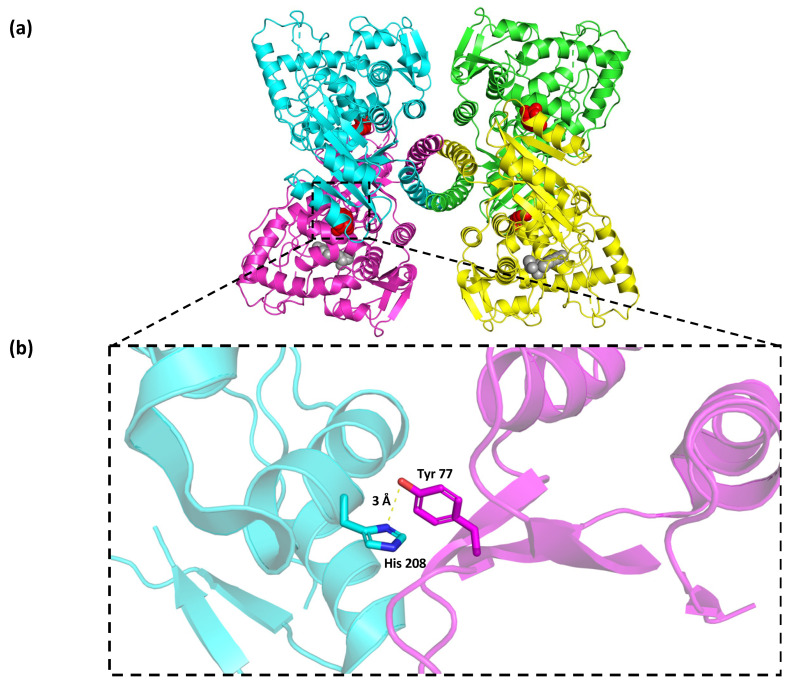
(**a**) Tetrameric phenylalanine hydroxylase functional unit. (**b**) Location of Tyr 77 residue in Wt PAH and its surroundings. The different colors in the figure of PAH enzyme denote the four different subunits that constitute the homotetramer.

According to the crystallographic structure of the PAH functional unit (Figure 3a), the in silico protein modeling for the Wt enzyme revealed that the Tyr 77 residue from chain A was 3 Å away from the residue His 208 from chain B, and these residues interacted through two hydrogen bonds and other non-bonded contacts (Figure 3b).

**Figure 4:**
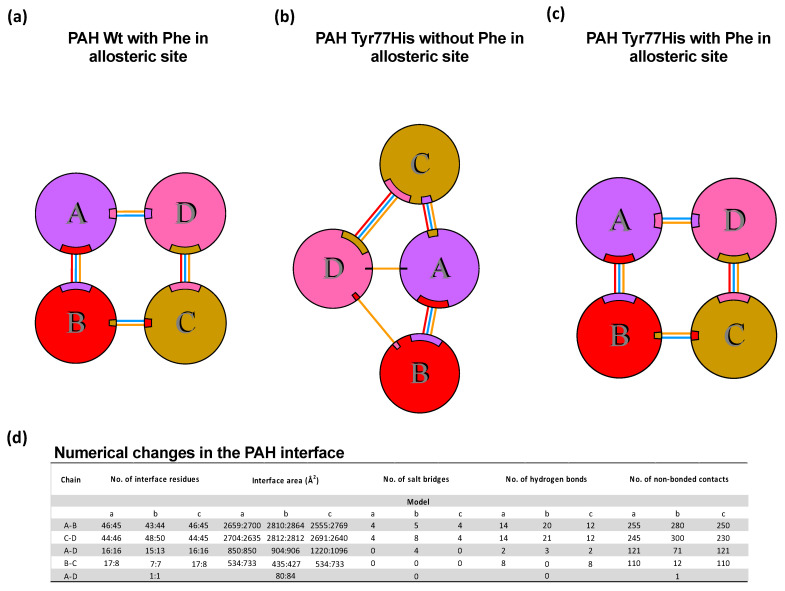
Interaction of PAH subunits in Wt PAH and the Tyr77His variant, with and without Phe at the allosteric site. (**a**) Spatial arrangement and interactions of subunits of Wt PAH when Phe is present in the allosteric site. (**b**) Spatial arrangement and interactions of subunits of p.(Tyr77His) PAH variant without Phe in the allosteric site. (**c**) Spatial arrangement and interactions of subunits of p.(Tyr77His) PAH variant when Phe is present in the allosteric site. (**d**) List of numerical changes in the interface area and the type of interactions in the PAH interface, depending on the type of enzyme (Wt or p.(Tyr77His) variant) and the presence or absence of Phe at the allosteric site. The predicted minimal changes in the interactions among the PAH subunits in the presence of Phe support the benign characteristics of the p.(Tyr77His) variant. The different colors in the figure of PAH enzyme denote the four different subunits that constitute the homotetramer.

The functional unit of PAH in humans is the tetramer. Our PDBsum analysis showed that, in the Wt PAH tetramer, chain A interacted with chains B and D, chain B interacted with chains A and C, chain C interacted with chains B and D, and chain D interacted with chains A and C. The formed interactions included salt bridges, hydrogen bonds, and other weaker non-bonded electrostatic contacts (Figure 4a). In the absence of Phe at the allosteric site, substitution of the Tyr residue at 77th position for His would lead to the complete reorganization of the PAH tetramer, including alterations to the interaction between chains A and C, the interface area, and the number of residues that establish the interfaces (Figure 4b). Conversely, in the presence of Phe at the allosteric site, this substitution of Tyr by His did not disturb the general tetramer organization, the number of interface residues, or the number of salt bridges (Figure 4c), although there were changes in the interface area involving several hydrogen bonds and several non-bonded contacts (Figure 4d).

**Figure 5:**
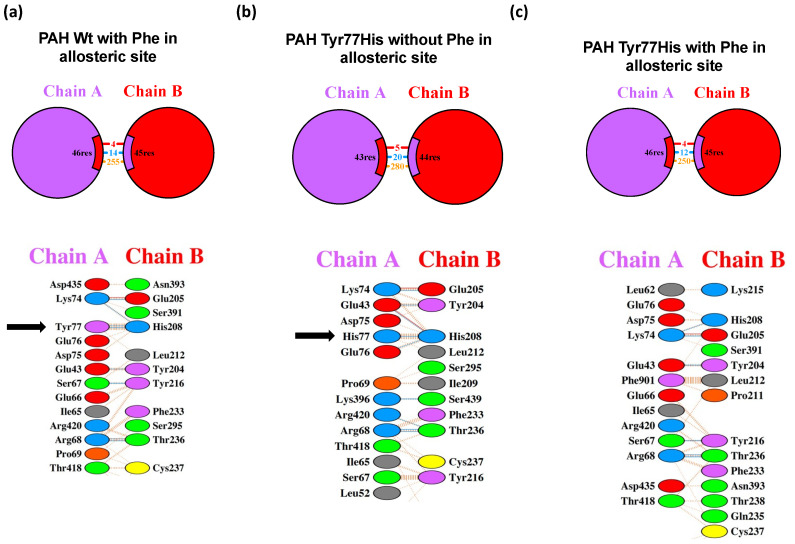
The close interface interactions of Tyr 77 in Wt PAH and p.(Tyr77His) PAH variant (arrows), with and without Phe at the allosteric site. (**a**) Detailed interactions of Tyr 77 in Wt PAH. The interactions of the p.(Tyr77His) variant are remarkably different in the absence (**b**) or presence (**c**) of Phe at the allosteric site.

The detailed PDBsum analysis showed that the Wt PAH Tyr 77 residue from chain A interacted with His 208 from chain B, while the latter residue established contacts with Lys 74, Asp 75, and Glu 76 from chain A (Figure 5a). Compared with Wt PAH, in the absence of Phe at the allosteric site, the substitution of Tyr by His at position 77 would cause only the loss of one salt bridge with the His 208 residue from subunit B; His 208 would still interact with Lys 74, Asp 75, and Glu 76 from subunit A and form a new interaction with Glu 43 (Figure 5b). When the p.(Tyr77His) variant was modeled in the PAH crystal with Phe at the allosteric site, the interaction of the substituted His 77 was lost, but His 208 still interacted with Lys 74, Asp 75, and Glu 76 and retained the newly established interaction with Glu 43 (Figure 5c).

**Figure 6:**
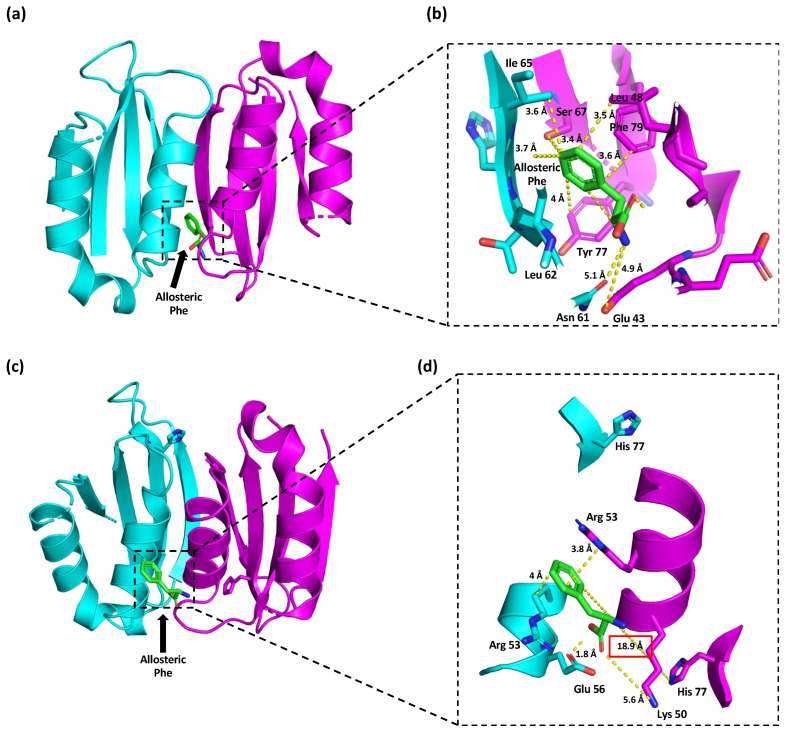
Interactions formed with and without Phe at the allosteric site of PAH. Interactions of Tyr 77 in Wt PAH (**a**,**b**) and those established with its substitution by His residue (**c**,**d**). With the substitution, allosteric Phe is moved to 14.9 Å away from His 77 (**d**). The colors in the figure denote the different subunits.

Regarding the position of Phe at the allosteric site, analysis of the crystallographic structure of the Wt PAH regulatory domain (PDB code: 5FII) showed that allosteric Phe interacted with Glu 43, Ser 67, Tyr 77, and Phe 79 from chain A, which were located at 4.9 Å, 3.4 Å, 4 Å, and 3.6 Å from the allosteric Phe, respectively (Figure 6a,b). Allosteric Phe also interacted with Asn 61, Leu 62, and Ile 65 from chain B at distances of 5.1 Å, 3.7 Å, and 3.6 Å, respectively. The colors in the figure denote the different subunits. 

In silico mutagenesis was performed on this regulatory domain to visualize the potential structural changes induced by the p.(Tyr77His) variant. The Phe molecule was then docked in this structure (Figure 6c). The best-ranked position of allosteric Phe was found at 18.9 Å from the substituted His 77. This Phe molecule also interacted with Lys 50 and Arg 53 from chain A and with Arg 53 and Glu 56 from chain B at distances of 5.6 Å, 3.8 Å, 4 Å, and 1.8 Å, respectively (Figure 6d).

## 4. Discussion

We herein present a newborn female patient who was born in Mexico from migrant Cuban parents and presented mild HPA conditioned by a previously unreported compound heterozygous *PAH* genotype involving two variants previously not described in the Mexican population [14]. While the p.(Tyr77His) variant had not been previously reported in the Cuban population, the “Celtic” or c.1222C>T or p.(Arg408Trp) variant was described with an allelic frequency of 5.3% in a Cuban cohort [29]. The two variants were previously registered in BioPKUdb [12], but this is the first description of the HPA phenotype conditioned by this compound heterozygous genotype.

In everyday clinical practice, the type of HPA/PKU must be clearly defined to prescribe proper treatment and medical follow-up. In severe forms with very high Phe blood levels, immediate treatment involving restriction of dietary Phe and limitation of breastfeeding is clearly indicated [30]. However, cases showing borderline Phe values indicative of mild or benign HPA can raise the question of “to treat or not to treat?” [2,10]. In such cases, *PAH* genotyping can be usefully interpreted in the biochemical and clinical context of the patient. For this reason, our confirmatory HPA diagnostic work-up includes genotyping.

The p.(Tyr77His) PAH variant was first reported by Ohlsson et al. in 2016 [16] as part of the mutational *PAH* spectrum in the Swedish population. It was predicted as a disease-causing allele in BioPKUdb [12], as supported by in silico evaluation with PolyPhen-2, PROVEAN, and Mutation Taster. Clinically, this c.229T>C or p.(Tyr77His) variant was associated with mild PKU (500 and 1200 μM of Phe at newborn screening) [12,16]. However, Ohlsson et al.’s report described only the variant, not the genotype, which precludes any comparison between their findings and our present results.

Due to no previous findings on residual activity and limited information regarding the HPA phenotype conditioned by p.(Tyr77His), we aimed to perform structural and PDBsum analyses of the resulting mutant PAH enzyme. The functional unit of PAH as well as other hydroxylases, such as tyrosine and tryptophan hydroxylases, are mainly tetrameric. However, homodimeric and homotetrameric forms of PAH exist in equilibrium [31]. PAH comprises an N-terminal regulatory domain (residues 1 to 142), a catalytic domain (residues 143–410), and an oligomerization domain (residues 411–452) [32]. Our structural analysis of the p.(Tyr77His) variant revealed that it would cause the loss of only one salt bridge (between Tyr 77 and His 208), and its remaining interactions would be unchanged (Figure 5b). This substitution would also provoke a relocation of allosteric Phe, which would be found 18.9 Å from His 77 (Figure 6c,d), whereas it is positioned only 4 Å from Tyr 77 in Wt PAH (Figure 6a,b). Thus, we expect that the mutant enzyme would retain most of its original function and, therefore, compensate for the effect of the more severe second allele p.(Arg408Trp). These structural predictions are consistent with the p.(Tyr77His) variant being associated with mild PKU even though the involved residue is located in the regulatory PAH domain [16,33]. However, further in vitro functional studies, such as the determination of enzymatic kinetics, as well as the estimation of the residual activity conferred by this variant are needed to establish its correlation with the predicted in silico structural changes.

The second variant found in our patient, c.1222C>T or p.(Arg408Trp), is located at the catalytic site and is a well-known allele; it has 2% residual enzymatic activity and conditions classic PKU [28] (Table 1). In functionally hemizygous patients (null/missense paired alleles), the less severe of the two PAH variants reportedly determines the disease severity [34,35]. Thus, the Celtic variant seems unlikely to contribute to the mild HPA phenotype observed in the patient, supporting our proposal that c.229T>C or p.(Tyr77His) could be considered a milder *PAH* allele.

After the diet intervention, our patient showed good blood Phe control. This suggests that she had certain residual PAH activity. The benign characteristics and good clinical and biochemical outcomes of this patient do not support the instauration of pharmacological treatment with sapropterin [10,11].

Population migration is a well-recognized phenomenon that can shift the epidemiologic landscape of genetic diseases. Some illustrative examples include the notorious increase in the incidence of Hb E/β-thalassemia and β-thalassemia major in California between 1990 and 2000, which was attributed to the rise in Asian immigration [36], and the increase in the incidence of galactokinase deficiency in Germany from 1992 to 1999, which was attributed mainly to the influx of Bosnian refugees [37]. In 2020, an estimated 11,186,000 individuals were living in Mexico as immigrants. However, from 2021 to 2022, Mexico experienced a 44% increase (nearly 445,000 individuals) in irregular immigration, mainly from Central American countries, including Cuba. In fact, the Cuban immigration flow was estimated to have increased 488% in Mexico from 2021 to 2022 [38]. The identification of uncommon diseases and genotypes associated with the immigration phenomenon, such as described herein, could be considered a warning that policymakers must stand ready to modify health policies, including NBS programs, to minimize the burdening impact of new potential emerging genetic diseases among the Mexican population [39].

## 5. Conclusions

We herein describe a previously unreported compound heterozygous *PAH* genotype identified in a mild HPA-affected newborn of Cuban ancestry. Her clinical and biochemical phenotype, along with our in silico structural protein modeling, indicated that p.(Tyr77His) could be considered a milder HPA-causing allele. This amino acid change is predicted to cause a minimal structural disturbance to the protein interface that might even be attenuated by the allosteric effect of Phe. This report on the p.(Tyr77His) variant could help guide Phe dietary restrictions and medical follow-up for patients harboring this potential hypomorphic *PAH* allele.

## Figures and Tables

**Table 1:** Characteristics of the identified *PAH* variants [12].

Variant	c.229T>C or p.(Tyr77His)	c.1222C>T or p.(Arg408Trp) [rs5030858]
Variant type	Substitution	Substitution
Coding effect	Missense	Missense
Gene region	Exon 3	Exon 12
Protein domain	Regulatory	Catalytic
Enzyme activity	Unknown	2%
Allelic phenotype value (APV)	Unknown	0
Worldwide allele frequency (gnomAD) [24]	Unknown	0.0008910
ClinVar [25]	Uncertain significance (ID: RCV003316903.1)	Pathogenic (ID: 577)
ACMG/AMP criteria for the interpretation of sequence variants [26,27]	Pathogenic IIIb (PS4, PM2, PM3, PP2, PP3, PP4)	Pathogenic IIb (PS3, PS4, PP1-S, PM1, PM2, PM3, PP1-M, PP1, PP2, PP3, PP4, PP5)
First reported	Ohlsson, et al., 2016 [16]	DiLella, et al., 1987 [28]

## Data Availability

The patient’s personal data are unavailable due to privacy and ethical restrictions. Other data related to this manuscript are available upon reasonable request.

## References

[1] Scriver C.R., Clow C.L. (1980). Phenylketonuria: Epitome of human biochemical genetics. N. Engl. J. Med..

[2] van Spronsen F.J., Blau N., Harding C., Burlina A., Longo N., Bosch A.M. (2021). Phenylketonuria. Nat. Rev. Dis. Primers.

[3] Blau N., Van Spronsen F.J., Levy H.L. (2010). Phenylketonuria. Lancet.

[4] Cleary M.A., Skeath R. (2019). Phenylketonuria. Paediatr. Child Health.

[5] Rausell D., García-Blanco A., Correcher P., Vitoria I., Vento M., Cháfer-Pericás C. (2019). Newly validated biomarkers of brain damage may shed light into the role of oxidative stress in the pathophysiology of neurocognitive impairment in dietary restricted phenylketonuria patients. Pediatr. Res..

[6] Borrajo G.J. (2021). Newborn screening in Latin America: A brief overview of the state of the art. Proc. Am. J. Med. Genet. Part C Semin. Med. Genet..

[7] García-Flores E.P., Herrera-Maldonado N., Hinojosa-Trejo M.A., Vergara-Vázquez M., Halley-Castillo M.E. (2018). Avances y logros del programa de tamiz metabólico neonatal (2012–2018). Acta Pediátr. Méx..

[8] Hillert A., Anikster Y., Belanger-Quintana A., Burlina A., Burton B.K., Carducci C., Chiesa A.E., Christodoulou J., Đorđević M., Desviat L.R. (2020). The genetic landscape and epidemiology of phenylketonuria. Am. J. Hum. Genet..

[9] Remec Z.I., Trebusak Podkrajsek K., Repic Lampret B., Kovac J., Groselj U., Tesovnik T., Battelino T., Debeljak M. (2021). Next-generation sequencing in newborn screening: A review of current state. Front. Genet..

[10] Pollitt R. (2012). Commentary: What degree of hyperphenylalaninaemia requires treatment?. J. Inherit. Metab. Dis..

[11] van Spronsen F.J. (2011). Mild hyperphenylalaninemia: To treat or not to treat. J. Inherit. Metab. Dis..

[12] Blau N. BIOPKU Data Base. http://www.biopku.org.

[13] Himmelreich N., Shen N., Okun J.G., Thiel C., Hoffmann G.F., Blau N. (2018). Relationship between genotype, phenylalanine hydroxylase expression and in vitro activity and metabolic phenotype in phenylketonuria. Mol. Genet. Metab..

[14] Vela-Amieva M., Alcántara-Ortigoza M.A., Ibarra-González I., González-del Angel A., Fernández-Hernández L., Guillén-López S., López-Mejía L., Carrillo-Nieto R.I., Belmont-Martínez L., Fernández-Lainez C. (2021). An Updated PAH Mutational Spectrum of Phenylketonuria in Mexican Patients Attending a Single Center: Biochemical, Clinical-Genotyping Correlations. Genes.

[15] Vela-Amieva M., Abreu-Gonzalez M., Gonzalez-del Angel A., Ibarra-Gonzalez I., Fernandez-Lainez C., Barrientos-Rios R., Monroy-Santoyo S., Guillén-López S., Alcántara-Ortigoza M. (2015). Phenylalanine hydroxylase deficiency in Mexico: Genotype–phenotype correlations, BH4 responsiveness and evidence of a founder effect. Clin. Genet..

[16] Ohlsson A., Bruhn H., Nordenström A., Zetterström R.H., Wedell A., von Döbeln U. (2016). The Spectrum of PAH Mutations and Increase of Milder Forms of Phenylketonuria in Sweden during 1965–2014. JIMD Reports.

[17] Hill D., Burnworth L., Skea W., Pfeifer R. (1982). Quantitative HPLC analysis of plasma amino acids as orthophthaldialdehyde/ethanethiol derivatives. J. Liq. Chromatogr..

[18] Flydal M.I., Alcorlo-Pagés M., Johannessen F.G., Martínez-Caballero S., Skjærven L., Fernandez-Leiro R., Martinez A., Hermoso J.A. (2019). Structure of full-length human phenylalanine hydroxylase in complex with tetrahydrobiopterin. Proc. Natl. Acad. Sci. USA.

[19] Patel D., Kopec J., Fitzpatrick F., McCorvie T.J., Yue W.W. (2016). Structural basis for ligand-dependent dimerization of phenylalanine hydroxylase regulatory domain. Sci. Rep..

[20] DeLano W.L. (2002). Pymol: An open-source molecular graphics tool. CCP4 Newsl. Protein Crystallogr..

[21] Laskowski R.A., Jabłońska J., Pravda L., Vařeková R.S., Thornton J.M. (2018). PDBsum: Structural summaries of PDB entries. Protein Sci..

[22] Krieger E., Joo K., Lee J., Lee J., Raman S., Thompson J., Tyka M., Baker D., Karplus K. (2009). Improving physical realism, stereochemistry, and side-chain accuracy in homology modeling: Four approaches that performed well in CASP8. Proteins Struct. Funct. Bioinform..

[23] Grosdidier A., Zoete V., Michielin O. (2011). SwissDock, a protein-small molecule docking web service based on EADock DSS. Nucleic Acids Res..

[24] Genome Aggregation Database (gnomAD). https://gnomad.broadinstitute.org/.

[25] ClinVar NCBI. https://www.ncbi.nlm.nih.gov/clinvar/.

[26] Richards S., Aziz N., Bale S., Bick D., Das S., Gastier-Foster J., Grody W.W., Hegde M., Lyon E., Spector E. (2015). Standards and guidelines for the interpretation of sequence variants: A joint consensus recommendation of the American College of Medical Genetics and Genomics and the Association for Molecular Pathology. Genet. Med..

[27] Kleinberger J., Maloney K.A., Pollin T.I., Jeng L.J.B. (2016). An openly available online tool for implementing the ACMG/AMP standards and guidelines for the interpretation of sequence variants. Genet. Med..

[28] DiLella A.G., Marvi J., Brayton K., Woo S.L. (1987). An amino-acid substitution involved in phenylketonuria is in linkage disequilibrium with DNA haplotype 2. Nature.

[29] Desviat L., Pérez B., Gutierrez E., Sanchez A., Barrios B., Ugarte M. (2001). Molecular basis of phenylketonuria in Cuba. Hum. Mutat..

[30] Acosta P., Matalon K.M., Phyllis B.A. (2010). Nutrition Management of Patients with Inherited Disorders of Aromatic Amino Acid Metabolism. Nutrition Management of Patients with Inherited Metabolic Disorders.

[31] Kobe B., Jennings I.G., House C.M., Michell B.J., Goodwill K.E., Santarsiero B.D., Stevens R.C., Cotton R.G., Kemp B.E. (1999). Structural basis of autoregulation of phenylalanine hydroxylase. Nat. Struct. Biol..

[32] Gersting S.W., Kemter K.F., Staudigl M., Messing D.D., Danecka M.K., Lagler F.B., Sommerhoff C.P., Roscher A.A., Muntau A.C. (2008). Loss of function in phenylketonuria is caused by impaired molecular motions and conformational instability. Am. J. Hum. Genet..

[33] Flydal M.I., Martinez A. (2013). Phenylalanine hydroxylase: Function, structure, and regulation. IUBMB Life.

[34] Guldberg P., Rey F., Zschocke J., Romano V., François B., Michiels L., Ullrich K., Hoffmann G.F., Burgard P., Schmidt H. (1998). A European multicenter study of phenylalanine hydroxylase deficiency: Classification of 105 mutations and a general system for genotype-based prediction of metabolic phenotype. Am. J. Hum. Genet..

[35] Regier DS G.C. Phenylalanine Hydroxylase Deficiency. https://www.ncbi.nlm.nih.gov/books/NBK1504/.

[36] Lorey F. (2000). Asian immigration and public health in California: Thalassemia in newborns in California. J. Pediatr. Hematol. Oncol..

[37] Reich S., Hennermann J., Vetter B., Neumann L.M., Shin Y.S., Söling A., MÖnch E., Kulozik A.E. (2002). An unexpectedly high frequency of hypergalactosemia in an immigrant Bosnian population revealed by newborn screening. Pediatr. Res..

[38] Perfil Migratorio de México Boletín Anual 2022 Organización Internacional para las Migraciónes (OIM). Organización de las Naciones Unidas (ONU) Migración. https://mexico.iom.int/sites/g/files/tmzbdl1686/files/documents/2023-03/Perfil%20Migratorio-%20Boletin%20Anual%202022%20%283%29.pdf.

[39] Aguilar Martinez P., Angastiniotis M., Eleftheriou A., Gulbis B., Manu Pereira M.D.M., Petrova-Benedict R., Corrons J.-L.V. (2014). Haemoglobinopathies in Europe: Health & Migration Policy Perspectives. Orphanet J. Rare Dis..

